# Spatio-Temporal Characteristics of Water Ecological Footprint and Countermeasures for Water Sustainability in Japan

**DOI:** 10.3390/ijerph191610380

**Published:** 2022-08-20

**Authors:** Yin Su, Qifang Zheng, Shenghai Liao

**Affiliations:** 1 College of Eco-Environmental Engineering, Guizhou Minzu University, Huaxi District, Guiyang 550025, China; 2 Faculty of Environmental Engineering, The University of Kitakyushu, 1-1 Hibikino, Wakamatsu-ku, Kitakyushu 808-0135, Japan

**Keywords:** water resources, ecological footprint, carrying capacity, ecological footprint accounts, water sustainability

## Abstract

Water-related problems are mostly caused by water imbalances between supply and demand. This study adopts the ecological footprint method to conduct an empirical study on the sustainable utilization of water resources in Japan. According to the basic principles and calculation methods of water ecological footprint (WEF), the characteristics of Japan’s water ecological footprint were investigated from the time and space dimensions, and a comparative analysis was made with the water ecological footprint of China. The results show that: from 1980 to 2020, the total water ecological footprint in Japan showed a downward trend in both the traditional account and pollutant account, and its spatial pattern was characterized by the relation that the higher the urbanization rate, the larger the water ecological footprint. In terms of water ecological footprint efficiency, Japan’s agricultural water ecological footprint efficiency was the lowest, and the domestic water ecological footprint efficiency was the highest. The water resources policies and measures that Japan and other developing countries should take to ensure the sustainability of water resources were analyzed separately.

## 1. Introduction

Water is a precious and irreplaceable resource supporting our agriculture, industry, manufacturing, and other daily life activities [1]. However, this precious water is now a limited and valuable resource rather than being abundant [2,3]. Although the Earth is known as the “water planet”, the freshwater available to people only accounts for about 2.5% of the planet’s total water. The spatial distribution of available water resources is uneven, resulting in some water-rich areas and some water-deficient areas [4]. In order to balance people’s demand for water in different regions, scholars have been focusing on the demand side of water. Water footprint [5] is an outstanding method, which is mainly based on the concept of virtual water, considering the water footprint inside and outside a region, and evaluating water scarcity along the supply chain [6]. Much of the existing water footprint research quantifies the water footprint of a region or the water contained in a specific product [7,8,9,10]. In fact, most of the international virtual water trade is related to the food trade [9,11]. The import of virtual water may bring negative effects such as a decrease in food self-sufficiency [12,13]. On the other hand, the export area consumes internal water resources due to the export of virtual water. Water resources in exporting regions are not inexhaustible, and some regions that export large quantities of water-consuming products have experienced severe water shortages in some months although these regions are relatively humid [14,15]. With the acceleration of economic globalization, water sustainability is no longer just a regional issue, but a worldwide issue [16]. Achieving water sustainability in some regions cannot come at the expense of water resources in other regions. Furthermore, in the context of global climate change, the challenge of water sustainability is even more acute [17,18,19]. No country can rely on virtual water trade for its own water security interests. Therefore, regardless of whether a region is water-scarce or water-rich, it is particularly important to address the issue of water sustainability from both supply and demand perspectives.

Ecological footprint [20,21,22] is defined as an area of biological productivity that provides humanity with everything it needs: fruits and vegetables, fish, wood, fibers, absorption of carbon dioxide from fossil fuel use, and space for buildings and roads [23]. The concept of water ecological footprint (WEF) is derived from the ecological footprint. Through the WEF method, the amount of water resources consumed by humans on the demand side is converted into the biological productivity of water resources, and the level and extent of sustainable development of regional water resources can be directly estimated by comparing with the ecological carrying capacity of water on the supply side [24,25]. The required water production area is called water ecological footprint, and the available water production area is called water ecological carrying capacity (WEC) [26,27]. The WEF method considers water imbalance issues from both the water supply and demand sides. Some scholars [28,29] evaluated the balance of water supply and demand with the WEF method and analyzed the relationship between supply and demand of water resources, ecological security, and water resources sustainability. The previous WEF model only accounted for the ecological footprint of water consumption and did not consider the ecological footprint of water pollution [30]. Li et al. [31] and Jin et al. [32] carried out theoretical innovation and empirical analysis of WEF from the aspects of water quantity and water quality.

Although there are different degrees of improvement in theory and application, it can be seen that there are still some gaps in previous research based on WEF. First of all, in some studies, the yield factor is treated as a constant [33,34]. Since the yield factor is determined by the average annual water resources and regional area, the total water resources change with the annual rainfall, and the yield factor also changes year by year. If the yield factor is constant, the WEC will be inaccurate. In order to ensure the accuracy of the yield factor in the calculation process, this paper calculates the yield factor of each year separately. In addition, most studies focus on water resource management based on the WEF model at the basin scale rather than administrative regions [35]. At present, research works either focus on the temporal evolution of WEF [36] or the spatial pattern of WEF [37], and there is a lack of research on the spatiotemporal evolution of WEF in a certain region. Meanwhile, focusing on a single case study of WEF ignores the comparative analysis of WEF. Due to the differences in water resource endowment, supply and demand structure, and water resource utilization efficiency, there are significant differences in the sustainability of water resources in different regions. These differences are the basis for regional comparative studies and the basis for studying water ecological footprints in different regions. Most of the previous WEF studies such as the calculation of water ecological footprint in Shenyang city [38] and the sustainable utilization of water resources in Liaoning province [39] have focused on underdeveloped regions, but few reports on the temporal and spatial evolution of WEF in developed regions. In addition, few relevant studies have deeply analyzed and compared the differences in WEF between less-developed and developed regions. With the development of globalization, the connection between all parts of the world is getting closer and closer, and it is very necessary to compare the water ecological footprint between different regions, especially the comparison between developed and underdeveloped regions. Therefore, in order to fill the gaps, the main innovations of the paper lie in the following points:

(1) This paper conducts a spatiotemporal analysis of the WEF using long-term statistical data and Japanese administrative region data.
(2) To avoid the weakness of the yield factor constant, this paper calculates the yield factor for each year separately.
(3) This study makes a comparative analysis of the WEF in developed and developing countries.

In the study, we adopt the WEF model to evaluate the water sustainability and efficiency in Japan, a developed country, in terms of water resource demand and supply. The structure of the article is arranged as follows: First, the characteristics of Japan’s WEF are analyzed from the perspective of time and space. Second, Japan’s water sustainability and water efficiency are evaluated using water resource sustainability indicators. Then, the differences in the WEF of Japan and China are compared and analyzed. Finally, the countermeasures and suggestions for the sustainability of water resources in developed and underdeveloped regions are put forward.

## 2. Material and Methods

### 2.1. Study Area and Data Sources

**Figure 1:**
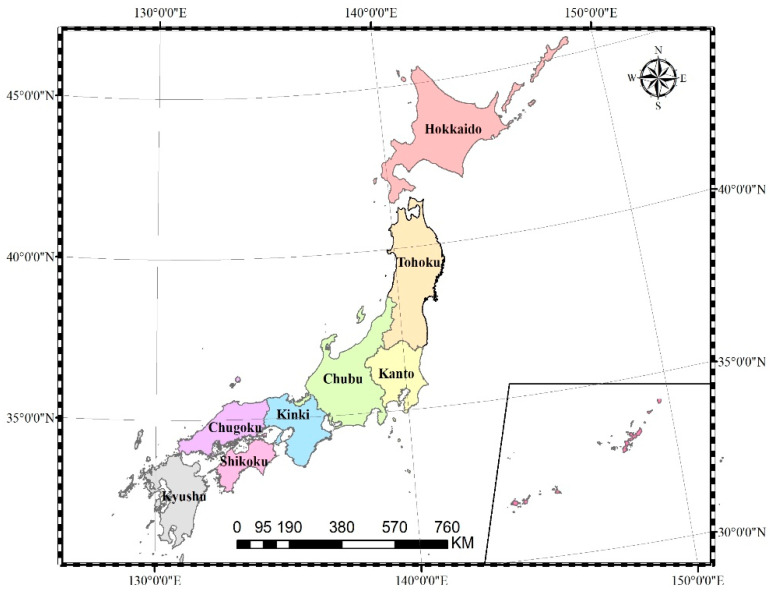
The administrative region of Japan.

Japan has a total of 6852 islands off the coast of East Asia and the Pacific. The area is about 378,000 km^2^ (Table 1). The country includes all islands and lies between latitude 24° to 46° north and longitude 122° to 146° east (Figure 1). The rivers in Japan are short, steep, and fast. Furthermore, the ratio between normal flow and flow during storms is very large. Japan’s precipitation (1718 mm) is nearly double the world average (943 mm), but the per capita precipitation (5114 m^3^) is only a quarter of the world average (21,796 m^3^). From the perspective of available water resources, Japan cannot be regarded as a water-rich country.

Data for the administrative division areas and the population were all obtained from the statistical handbook of Japan [40]. For water-related data, the total consumption of water resources, the sector account’s water consumption, and the total quantity of water resources were derived from the report on the current status of water resources in Japan [41,42,43,44,45]. Water pollutants emissions data were derived from the report of a comprehensive survey on water pollutant release [46,47,48,49,50].

### 2.2. Water Ecological Footprint Model

#### 2.2.1. The Total WEF

The traditional WEF model converts the water resource to a global standard biological productive land area after the fossil energy land and the arable land, the forest land, the pastureland, the construction land, and the sea space [24]. However, the model only considers the WEF of the agricultural, industrial, and domestic accounts, and does not consider the WEF of the pollution account. In this paper, the WEF model is divided into two accounts, one is the WEF of the traditional account and the other is the WEF of the pollutant account. The following are the formulas:

(1) $$WEF_{tot}=WEF_{tra}+WEF_{pol}$$

where *WEF_tot_* (ghm^2^) is the total water ecological footprint; *WEF_tra_* (ghm^2^) is the *WEF* of the traditional account; *WEF_pol_* (ghm^2^) is the WEF of the pollutant account.

#### 2.2.2. The WEF of Traditional Account

The traditional water use account includes agricultural water use, industrial water, use, and domestic water use. Thus, WEF contains the agricultural WEF, the industrial WEF, and the domestic WEF, which are calculated by the following formulas:

(2) $$WEF_{tra}=r_{w}*(W/p_{w})$$

$$W=W_{i}+W_{d}+W_{ag}$$

where *W* (m^3^) is the total water use; *W_i_* (m^3^) represents the industrial water use; *W_d_* (m^3^) represents the domestic water use; *W_a__g_* (m^3^) represents the agricultural water use. *r_w_* is the equivalence factor of the water resource. *p_w_* (m^3^/ghm^2^) is the global average yield of the water resource, and ghm^2^ is the abbreviation of the ecological footprint unit global hm^2^.

#### 2.2.3. The WEF of Pollutant Account

Generally speaking, sewage contains many forms of pollutants, and the most critical pollutant determines the WEF of the pollutant account. According to a relevant study [51], the paper selects total nitrogen (TN), total phosphorus (TP), and chemical oxygen demand (COD) as the representative indicators to measure the impacts of water pollution on water ecological quality. As these three kinds of indicators have obvious overlap in water quality impact, the largest WEF among them is taken as the final WEF of the pollutant account [51]. The formulas are:

(3) $$\begin{array}{l} WEF_{pol}=\max(WEF_{TN},WEF_{TP},WEF_{COD})\\ \left\{\begin{array}{l} WEF_{TN}=r_{w}*(U_{TN}/p_{N})\\ WEF_{TP}=r_{w}*(U_{TP}/p_{P})\\ WEF_{COD}=r_{w}*(U_{COD}/p_{COD}) \end{array}\right. \end{array}$$

where *WEF_TN_* (ghm^2^) is the *WEF* of total nitrogen; *WEF_TP_* (ghm^2^) is the *WEF* of total phosphorus; *WEF_COD_* (ghm^2^) is the *WEF* of chemical oxygen demand; *U_TN_* (t) is total nitrogen emissions of the study region; *U_TP_* (t) is total phosphorus emissions of the study region; *U_COD_* (t) is total chemical oxygen demand emissions of the study region; *P_N_* (t/ghm^2^) is the average TN absorption per unit area; *P_P_* (t/ghm^2^) is the average TP absorption per unit area; *P_COD_* (t/ghm^2^) is the average COD absorption per unit area.

#### 2.2.4. The WRCC

The water resource ecological carrying capacity (WRCC) is calculated as:

(4) $$WRCC=N\;*\;wrcc=0.88\;*\;\psi_{w}*\;r_{w}*\;(Q/p_{w})$$

where, *WRCC* (ghm^2^) is the total water resource carrying capacity, *wrcc* (ghm^2^/capita) is the water resource carrying capacity per capita, *ψ_w_* is the regional yield factor, and *ψ_w_* = *ψ*/*ψ_g_* and *ψ* is the quantity of the water production per area; *ψ_g_* is the quantities of the global water production per area. *Q* (m^3^) is the regional total amount of water resource, and it is a normal practice to allocate 12% of the available supply land to protect the local biodiversity [21,52].

When the WRCC is less than the WEF in a region, it goes into water ecological deficit (WED) and indicates that human pressure on water exceeds the water sustainability; when the WRCC is more than the WEF in a region, it goes into water ecological surplus (WES), which means its regional water utilization is sustainable; and when WRCC = WEF, it means that the regional water resource is in balance.

#### 2.2.5. The WEF Efficiency

To effectively measure the real water use per unit GDP, in this paper we constructed a WEF efficiency index. The index is simply the ratio of WEF to GDP, and it indicates the amount of WEF produced per unit GDP. The efficiency index can be calculated with Equation (5):

(5) $$g=\frac{WEF}{GDP}$$

Here, *g* represents the WEF efficiency. The greater the value, the lower the *g*, and vice versa.

### 2.3. Parameters Treatment

Just like the ecological footprint model, the main parameters in the WEF include the equivalence factor and the yield factor of water resources. According to the analysis of Huang et al. [24], this paper adopts World Wide Fund for Nature Living Planet Report (WWF 2002) to determine the equivalence factor of water resource as 5.19. The global average yield of water resource is 3140 m^3^/ghm^2^.

Based on the environmental water quality standards (EQS) [53] of Japan, the upper limit of chemical oxygen demand (COD), total nitrogen (TN) and total phosphorus (TP) is 8 mg/L, 1 mg/L, and 0.1 mg/L, respectively. Correspondingly, the values of *P_COD_*, *P_TN_*, and *P_TP_* are 0.02512 t/ghm^2^, 0.00314 t/ghm^2^, and 0.00031 t/ghm^2^, respectively. The upper limit of COD, TN, and TP refers to the maximum allowable value in water that can maintain ecological service functions without affecting the daily life of people, whether in rivers, lakes, or coastal waters. On the contrary, if the content of COD, TN, and TP in water exceeds the maximum allowable value, the ecological service function of the water body will decline or be lost.

## 3. Results

### 3.1. Temporal Characteristics of the WEF in Japan

#### 3.1.1. The Total WEF

**Figure 2:**
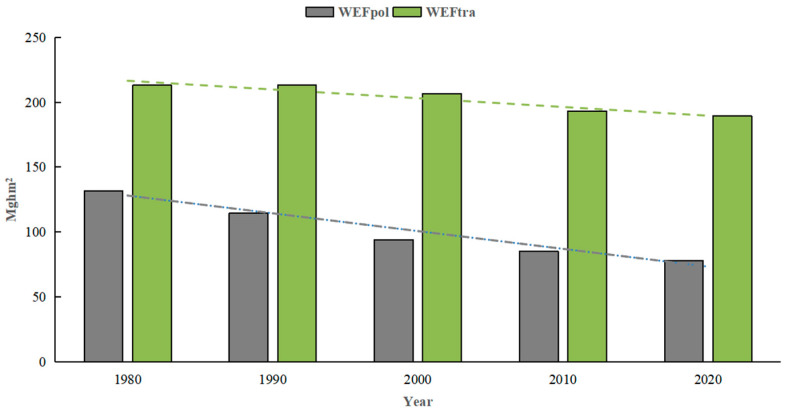
Transition of the total WEF in Japan during 1980–2020.

Based on the constructed WEF framework above, the total WEF of Japan consists of two parts: the WEF_tra_ and the WEF_pol_. We explored the temporal characteristics of the WEF accounts in Japan. As shown in Figure 2, it illustrated the total WEF in Japan decreased from 344.86 Mghm^2^ in 1980 to 267.50 Mghm^2^ in 2020, a decrease of 22.4%. Simultaneously, the WEF_tra_ experienced a decrease from 213.04 Mghm^2^ to 189.39 Mghm^2^, and the WEF_pol_ also dropped from 131.82 Mghm^2^ to 78.11 Mghm^2^, with a decrease of 11.1% and 40.7%, respectively. Although the proportion (40%) of the WEF_pol_ in the total WEF was lower than that of the WEF_tra_, the WEF_pol_ declined faster than that of the WEF_tra_ during the study period.

#### 3.1.2. The WEF of Traditional Account

**Figure 3:**
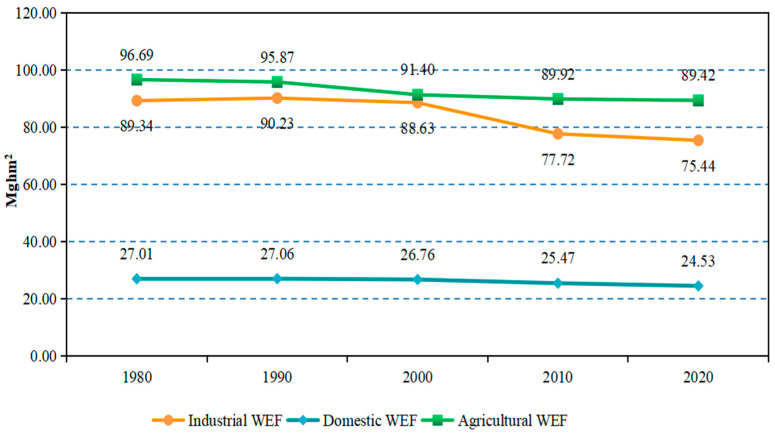
The WEF of traditional account in Japan during 1980–2020.

As shown in Figure 3, it illustrated the total traditional WEF in Japan experienced a steady decrease from 213.0 Mghm^2^ in 1980 to 189.4 Mghm^2^ in 2020, while the sub-accounts showed different trends. The agricultural WEF was the highest, followed by the industrial WEF, and finally the domestic WEF. The agricultural WEF had been showing a downward trend, from 96.69 M ghm^2^ in 1980 to 89.42 M ghm^2^ in 2020, a decline rate of 7.5%. In contrast, the industrial WEF initially increased slightly and began to decline rapidly since 2000, with a decline rate of about 15%, while the domestic WEF fluctuated little, presenting a slow decreasing tendency.

Water reuse history in Japan started in the 1980s in response to severe drought and increased water demand caused by rapid urbanization and economic growth [54]. Japan’s water reuse technology was first applied in agriculture to resist the impact of drought on agriculture, so the agricultural WEF first showed a downward trend. By 2000, the reuse of non-drinking water was mainly used to compensate for industrial water, which confirmed that the industrial WEF has dropped sharply since 2000 in Figure 3. With the maturity of water reuse technology and equipment, especially in urban water use, Japan has also gradually implemented the reuse of reclaimed water, which is why the domestic WEF has gradually and steadily decreased.

#### 3.1.3. The WEF of Pollutant Account

**Figure 4:**
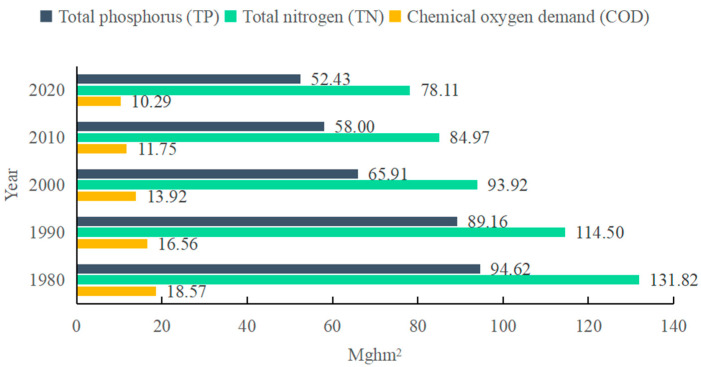
The WEF of pollution account in Japan during 1980–2020.

According to Formula (3), the WEF of the pollutant account was calculated. Due to the apparent overlap of the three pollutants in the environmental impact, the largest WEF among the three pollutants was used as the final WEF of pollutant account. From Figure 4, we can see that in Japan’s water pollutant accounts, the WEF of TN was the largest, followed by the WEF of TP, and the lowest the WEF of COD. Therefore, we chose the WEF of TN as the final WEF of the pollutant account in Japan. This means that in Japan’s water pollutants, TN has the greatest impact on the water quality, and it needs to consume more fresh water to dilute, that is to say, it needs to occupy more water-productive areas. Therefore, reducing TN pollution in Japan can greatly improve water pollution issues and reduce the WEF of the pollutant account.

At the same time, the WEF of the pollutant account in Japan experienced a steady decrease among the WEF of COD, the WEF of TN, and the WEF of TP with a decreasing rate of 44.6%, 40.7%, and 44.6%, respectively. The main reason is that the Total Pollutant Load Control System (TPLCS) was applied to reduce the total amount of pollution loads including industrial wastewater and domestic sewage in Japan in the 1980s. Through these control measures, the deterioration of water quality has been suppressed, and the water quality has been improved since then.

### 3.2. Spatial Characteristics of the WEF in Japan

#### 3.2.1. The WEF of Traditional Account

**Figure 5:**
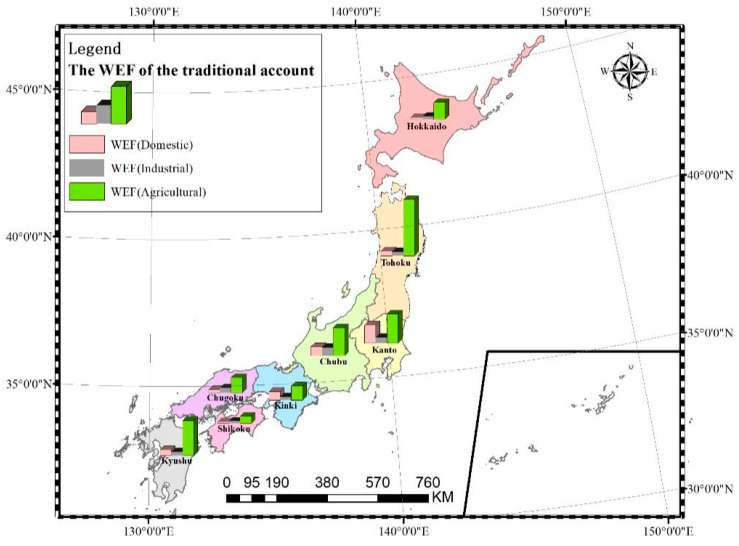
The WEF of traditional account in different administrative divisions of Japan in 2020.

In order to uncover the spatial characteristics of WEF in Japan, we also analyzed the WEF from the perspectives of the traditional account and pollutant account in 2020. In Japan, “eight regional divisions” are often used. From north to south, the regions are Hokkaido, Tohoku, Kanto, Chubu, Kinki, Chugoku, Shikoku, and Kyushu region. In traditional account, the WEF of the agricultural sector in Tohoku was the largest at 25.79 M gha and the smallest at 3.47 M gha was in Shikoku (Figure 5). This means that agricultural water consumption was the main driving force of the traditional account in Tohoku. In the WEF of the industrial sector, Chubu accounted for the highest proportion, followed by Kanto. The WEF of the domestic sector in Kanto was the largest. The WEF of industrial and domestic sectors was higher in Kanto because it is the political, economic, and cultural center of Japan. It has the highest population density and the largest industrial district, the Keisei Industrial Zone, in Japan. For whichever agricultural account, industrial account, or domestic account in the traditional account, the WEF of Shikoku was the smallest. It is mainly because Shikoku has the smallest area and the smallest population.

#### 3.2.2. The WEF of Pollutant Account

**Figure 6:**
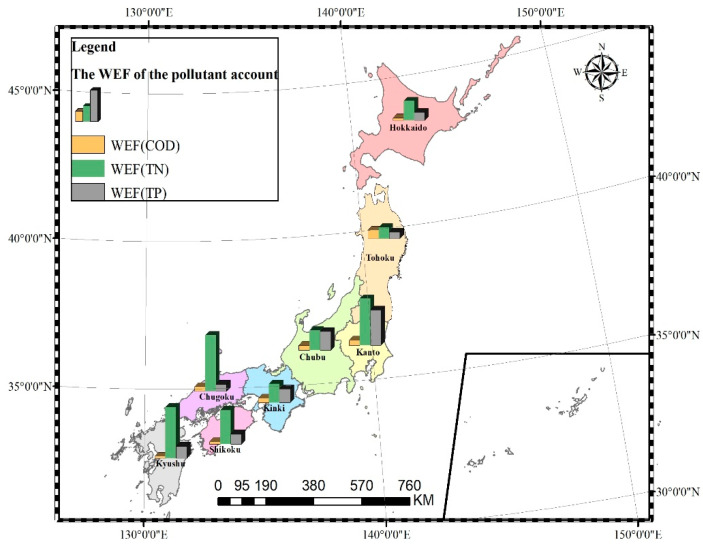
The WEF of pollution account in different administrative divisions of Japan in 2020 (Unit: 1 M gha).

From Figure 6, we can see that in the pollutant account of each region, the WEF of TN was also the largest. Therefore, the WEF of TN is used as the final WEF of pollutant account in each region. Just as Figure 6 showed, the WEF of TN in Chugoku ranked firstly, reaching 41.47 M gha. The second largest was the Kyushu region, followed by the Kanto region. The WEF of TN in the Tohoku region was the smallest at 8.28 M gha. The WEF of TN in Chugoku was about five times that of the Tohoku.

### 3.3. Water Sustainability Evaluation

#### 3.3.1. WES or WED

**Figure 7:**
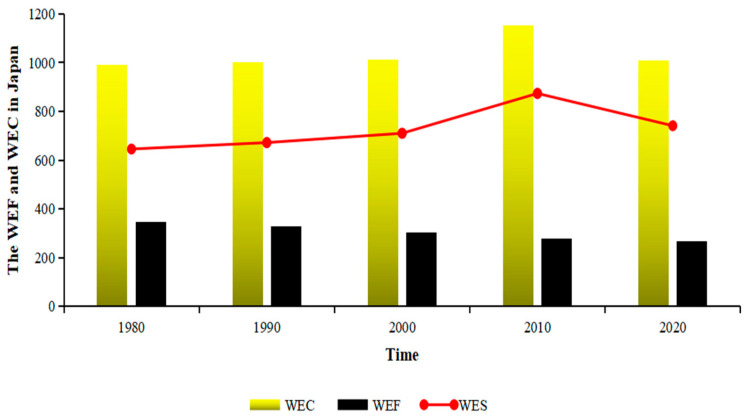
Changes of the WEF and WEC in Japan from 1980 to 2020 (Unit: 1 M gha).

In terms of time, when the WEC in a region is less than the demand for WEF, it goes into water ecological deficit (WED), and vice versa. The WED indicates that human pressure on water exceeds the local WEC in the region and that its regional water utilization is relatively unsustainable. On the contrary, water ecological surplus (WES) indicates that the regional WEC meets the water demand of current economic activity, and its regional water utilization is relatively sustainable. Figure 7 shows the total WEF, WEC, and WES for Japan from 1980 to 2020. The total WEF of Japan in 1980 was 345.32 M gha, decreasing to 267.27 M gha in 2020. Meanwhile, the WEC was 990.58 M gha during the period, resulting in the WES of 645.26 M gha in 1980 and 740.96 M gha in 2020. From Table 2 we found that the total WEF kept falling down no matter in the WEF of the traditional account or the WEF of the pollutant account. However, the proportion of the water traditional account was increasing from 61.7% in 1980 to 70.9% in 2020. Simultaneously, the proportion of the water pollutant account was decreasing from 38.3% in 1980 to 29.1% in 2020. That is to say, the WEF of the pollutant account drops faster than the WEF of the traditional account. The WES in Japan was so large that it not only met self-sufficiency but also was exported to other regions in a way of water production.

In terms of space, as can be seen from Table 3, the WEC of each region in 2020 was greater than the WEF, and different levels of WES existed in different regions. The WEC in the Chubu region of Honshu Island was the largest, and the WES was also the largest; the WEC in Chugoku was the smallest, and the WES was also the smallest. It shows that water resources in the Chubu region were relatively sufficient to meet all aspects of water use. It indicates that the water resources in Chubu were rich with great development potential, while the development potential in Chugoku was low. In the total WEF of all regions, the total WEF of the Kanto region was the largest at 58.67 M gha, which means the largest occupation of water. The main reason is that the Kanto region is Japan’s economic center and the most densely populated area. Simultaneously, the water quantity account and water quality account accounted for 41.3% and 58.7%, respectively. So, the water use intensity in the Kanto region in both water quantity and quality was roughly the same. Since Hokkaido’s economy and industry were dominated by tourism, with a small population size, the total WEF was the lowest at 24.57 M gha in Hokkaido.

#### 3.3.2. The WEF Efficiency

**Figure 8:**
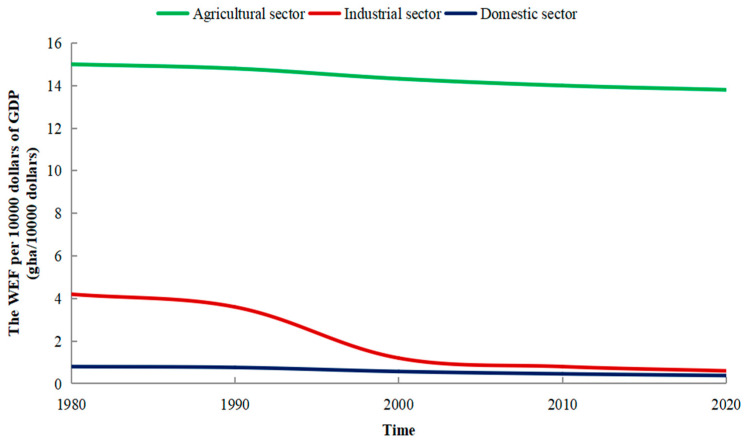
Changes in the WEF efficiency in Japan from 1980 to 2020.

The WEF efficiency index (Figure 8) was calculated as it is a comprehensive index of different economic sectors including the agricultural sector corresponding to agricultural water use, the industrial sector corresponding to urban industrial water use, and the domestic sector corresponding to municipal water use. It comprehensively reflects the regional water use efficiency. In terms of the agricultural sector, the WEF per ten thousand dollars GDP among the three sectors was the highest during 1980–2020, with a higher WEF level as well as a lower level of efficiency. The reason is that the proportion of the agricultural sector in Japan’s economic structure has decreased year by year, but the water consumption of the agricultural sector has not changed significantly. It means that the utilization rate of water resources in the agricultural sector was relatively low. In terms of the industrial sector, the WEF per ten thousand dollars GDP presented a degenerative trend annually. It had decreased six-fold from 4.2 gha/10,000 dollars in 1980 to 0.6 gha/10,000 dollars in 2020. The water use efficiency in the industrial sector has significantly improved. And in terms of the domestic sector, the WEF per ten thousand dollars GDP was basically stable and the value was less than 1 gha/10,000 dollars from 1980 to 2020. The value of the domestic sector remained at around 0.5 gha/10,000 dollars and has not changed much over the years. The domestic sector had a low demand for water resources, and the WEF was relatively small. In the GDP composition structure of Japan, the domestic sector accounted for a large proportion. Therefore, from the view of the WEF efficiency, the water use efficiency in the domestic sector was the highest. With the highest water use efficiency, the domestic sector should be vigorously developed.

## 4. Discussion

### 4.1. For Water Management in Japan

From the calculation formula of WEF, we can know that WEF is affected by the amount of water consumption, and the amount of water consumption is mainly affected by socioeconomic development and water resource utilization efficiency. In terms of the traditional water resources account, the WEF of the three major accounts decreased from 1980 to 2020, reflecting the decreasing demand for water resources in Japan’s social economy. The decline of WEF also verifies that Japan’s socioeconomic development has been stagnant for more than three decades. In terms of polluted water resources account, the WEF of COD is smaller than the WEF of TP, and the WEF of TP is smaller than the WEF of TN, indicating that the largest water pollutant in Japan is nitrogen pollution. Therefore, Japan should reduce nitrogen emissions to water bodies to reduce the WEF of pollutant account. Japan’s high WEC reflects the overall abundance of water resources in Japan. The WES is relatively large and has a strong positive correlation with the WEC, indicating that Japan has a great potential for sustainable development and utilization of water resources, and socioeconomic development will not be constrained by water resources. In terms of the WEF efficiency, the agricultural sector has the largest WEF per $10,000 of GDP. This means that the agricultural sector is the least water efficient. It is recommended that Japan save water by importing water-intensive alternatives from outside or applying advanced water-saving equipment to improve agricultural water-use efficiency.

### 4.2. For Water Management in Other Nations

As a developed country in Asia, the characteristics of water use and WEF in Japan are different from other developing countries in Asia. In order to better clarify the differences in water use between Japan and other developing countries and provide a reference for the development of water resources in other developing countries in Asia, we chose China as a typical developing country to compare with Japan. The water sustainability indicators of China and Japan during the same period were selected as the compared object, thus contributing to water resources management in developing countries. In terms of WES or WED, according to relevant literature [55], WEF in China exceeded WEC for the first time in 1980, and WED appeared. The results of this study show that the WES of Japan in 1980 was 645.26 M gha. Based on SUN’s research [56], by 2014, China’s WEF was 2579.14 M gha, WEC was 1652.82 M gha, and WED reached 926.32 M gha, while the results of the paper show that Japan’s WES increased from 645.26 M gha in 1980 to 873.57 M gha in 2010. In terms of the WEF efficiency indicator, referring to Tan’s research, the WEF per ten thousand dollars GDP in China was 17.88 gha (1 US dollar = 8.2784 RMB yuan) in 2000, while in Japan it was 14.32 gha for agriculture, 1.20 gha for industry and 0.57 gha for domestic production based on our results. A comparison of China and Japan can provide valuable policy insights for formulating appropriate water resource management strategies, especially for developing countries whose rapid economic development is constrained by water resources. First, developing countries like China should reduce WEF by promoting the development and application of reclaimed water technology, because it is well known that water recycling in Japan has increased rapidly since the 1980s, and reclaimed water is an important compensation for water supply. Secondly, the structure of water resource utilization needs to be optimized to ease the pressure on water ecology, and the efficiency of water resource utilization needs to be further improved.

In addition, there are some general recommendations. On the one hand, it is critical to improve the WEC. For water-scarce regions, researchers should clearly focus their efforts on reclaiming water technologies rather than finding traditional water sources. The cost of water transfer projects and uneven distribution of water resources determine that reclaimed water is an irreplaceable and necessary supplement for water supply. We all know that water availability is decreasing as demand increases for domestic, industrial, and agricultural uses. The conventional water sources can no longer meet the needs of urban settlements. As a result, more and more efforts are being made to employ various technologies to improve the reuse of urban and industrial wastewater, desalination of saline groundwater, agricultural drainage, and seawater. The government should allocate more research funding to support related water recycling activities to improve water availability. On the other hand, it needs to reduce the WEF as much as possible. From the characteristics of the water resource utilization structure, it can be seen that the main demand pressure comes from the development of industry and agriculture. Therefore, adjusting and upgrading the industrial structure is the key to reducing water consumption and improving water efficiency. In terms of water traditional accounts, the WEF in the agricultural sector is in most cases the largest, and agricultural water use efficiency is the lowest [57]. While the domestic sector has the smallest WEF and higher domestic water use efficiency. Therefore, for other water-scarce regions or countries, it is particularly necessary to make efforts to develop the domestic sector and adjust with a focus on the agricultural sector. In terms of water pollution account, with rapid economic development and urbanization, developing countries are facing increasingly serious water pollution. It is urgent to reduce water pollution and the WEF of water pollution account.

## 5. Conclusions

Based on the WEF model and the obtained data on water resource utilization in Japan, this paper improves the calculation method of yield factor so that the evaluation indicators will be more reliable and accurate. The traditional account and water pollution account of Japan’s WEF are calculated and analyzed from the two dimensions of time and space, and the sustainable use of water is evaluated using water sustainability indicators. Finally, the differences in the WEF of China and Japan are compared. Japan’s experience in water resource management has implications for many developing Asian countries that are currently facing major water challenges. We hope this will help provide valuable insights for policymakers to propose more sustainable water use policies that incorporate local realities. The main conclusions of this study are listed as follows:

1. During the study period, both the water traditional account and the water pollution account, Japan’s water ecological footprint showed a downward trend. The agricultural sector has the highest WEF in the water traditional account and the WEF of TN is the largest in the water pollution account.
2. The spatial distribution pattern of the WEF in Japan is that whether it is a traditional water account or a water pollution account, the WEF is concentrated in the industrial corridors along the Pacific coast. For example, Kanto, Chugoku, and Kyushu have the highest WEF because the urbanized areas are mainly along Japan’s Pacific coast from the Kanto region to Osaka and the Inland Sea (on both sides) and Fukuoka.
3. Proposing relevant policies. Japan should further improve the utilization efficiency of water resources, reduce the WEF of agriculture in traditional water accounting, and reduce the WEF of TN in water pollution accounting. For underdeveloped water-deficient areas, the industrial structure should be optimized, the efficiency of water resource utilization should be improved, and more feasible water cycle technologies should be adopted to achieve sustainable water development.

At the same time, we should also pay attention to the limitations of the paper. The parameters such as equivalence factor and yield factor in the WEF model are based on the supposition of substitutability between man-made and natural capital and change with study areas or years, ignoring the differences of the technological and socioeconomic influence on bio-productivity, which generally results in considerable deviations. Predictions of the water ecological footprint should also be considered to determine future trends in the water ecological footprint method. These are future research directions.

## Figures and Tables

**Table 1:** Basic overview of different administrative divisions in Japan.

Administrative Divisions	Population (K)	Total Quantity of Annual Mean Water Resources (0.1 G m^3^)	Area (M gha)	Regional Average Water Production er Unit Area (m^3^/gha)	Yield Factor
Hokkaido	5506	563	8.35	6745.99	2.15
Tohoku	11,710	868	7.95	10,913.43	3.48
Kanto	43,468	393	3.69	10,653.29	3.39
Chubu	55,529	853	5.55	15,361.34	4.89
Kinki	20,904	307	2.73	11,228.15	3.58
Chugoku	7563	328	3.19	10,275.37	3.27
Shikoku	3977	277	1.88	14,729.34	4.69
Kyushu	14,597	646	4.45	14,527.63	4.63
Japan	163,254	4235	37.79	11,205.27	3.57

**Table 2:** The total WEF and WES in Japan from 1980–2020 (Unit: 1 M gha).

	WEF			WES
	The WEF of Traditional Account	The WEF of Pollutant Account	Total	
1980	213.038	132.283	345.321	645.259
1990	213.154	115.428	328.582	671.678
2000	206.790	94.505	301.295	709.955
2010	193.104	85.554	278.659	873.571
2020	189.385	77.882	267.268	740.962

**Table 3:** The total WEF, WEC, and WES of different regions in 2020 (Unit: 1 M gha).

	WEC	WEF			WES
		The WEF of Traditional Account	The WEF of Pollutant Account	Total	
Hokkaido	79.969	10.165	14.400	24.565	55.404
Tohoku	199.457	29.785	8.281	38.065	161.392
Kanto	88.155	24.198	34.461	58.659	29.496
Chubu	275.897	20.727	14.638	35.365	240.532
Kinki	72.580	12.314	13.955	26.269	46.311
Chugoku	70.964	10.529	41.471	52.000	18.964
Shikoku	85.908	5.405	25.198	30.603	55.305
Kyushu	197.604	20.429	37.713	58.142	139.461

## Data Availability

The data that support the findings of this study are available from the corresponding author upon reasonable request.

## Nomenclature

*EF*	Ecological footprint
*WEFM*	Water ecological footprint model (gha)
*WEFA*	Water ecological footprint account
*WEF_wr_*	The WEF of water resources account(gha)
*WEF_we_*	The WEF of water environment account(gha)
*WEF*	Water ecological footprint (gha)
*WEC*	Water ecological carrying capacity (gha)
*WED*	Water ecological deficit (gha)
*WES*	Water ecological surplus (gha)
*WEF_TN_*	The WEF of total nitrogen(gha)
*WEF_TP_*	The WEF of total phosphorus(gha)
*WEF_COD_*	The WEF of chemical oxygen demand(gha)
*W*	Total water consumption (m^3^)
*W_i_*	The industrial water consumption(m^3^)
*W_d_*	The domestic water consumption(m^3^)
*W_p_*	The paddy field irrigation water consumption(m^3^)
*W_up_*	The upland field irrigation water consumption(m^3^)
*W_an_*	The animal husbandry water consumption(m^3^)
*r_w_*	The global balance factor of water resources
*p_w_*	The world average water yield factor (m^3^/hm^2^)
*ψ_w_*	The water resources yield factor
*Q*	The regional total amount of water resource (m^3^)
*TN*	Total nitrogen
*COD*	Chemical oxygen demand
*TP*	Total phosphorus
*U_TN_*	Total nitrogen emissions(t)
*U_TP_*	Total phosphorus emissions(t)
*U_COD_*	Total chemical oxygen demand emissions(t)
*P_N_*	The average TN absorption per unit area(t/gha)
*P_P_*	The average TP absorption per unit area(t/gha)
*P_COD_*	The average COD absorption per unit area(t/gha)
*WEPI*	The indicator of water ecological press
*WUEI*	Water use efficiency index(gha/ten thousand dollars)
*GDP*	Gross Domestic Product(dollar)
*TPLCS*	Total Pollutant Load Control System
*WWF2002*	World Wide Fund for Nature Living Planet Report 2002
*EQS*	Environmental water quality standard
